# Biological application of Compressed Sensing Tomography in the Scanning Electron Microscope

**DOI:** 10.1038/srep33354

**Published:** 2016-09-20

**Authors:** Matteo Ferroni, Alberto Signoroni, Andrea Sanzogni, Luca Masini, Andrea Migliori, Luca Ortolani, Alessandro Pezza, Vittorio Morandi

**Affiliations:** 1 Brescia University, Dept. of Information Engineering, Via Branze, 25123 Brescia, Italy; 2 CNR - National Institute of Optics, Via Valotti, 25123 Brescia, Italy; 3 CNR - Microelectronic and Microsystems Institute, via Gobetti 101, 40129 Bologna, Italy

## Abstract

The three-dimensional tomographic reconstruction of a biological sample, namely collagen fibrils in human dermal tissue, was obtained from a set of projection-images acquired in the Scanning Electron Microscope. A tailored strategy for the transmission imaging mode was implemented in the microscope and proved effective in acquiring the projections needed for the tomographic reconstruction. Suitable projection alignment and Compressed Sensing formulation were used to overcome the limitations arising from the experimental acquisition strategy and to improve the reconstruction of the sample. The undetermined problem of structure reconstruction from a set of projections, limited in number and angular range, was indeed supported by exploiting the sparsity of the object projected in the electron microscopy images. In particular, the proposed system was able to preserve the reconstruction accuracy even in presence of a significant reduction of experimental projections.

Electron Tomography (ET) is a prominent technique in the field of three-dimensional (3D) characterization in physical and life sciences, where the mass distribution of the sample is retrieved from projection-images taken at different angles. ET is primarily carried out in the Transmission Electron Microscope (TEM), operated in a number of imaging modes: Bright-Field, High-Angle-Annular-Dark-Field (HAADF) Scanning-TEM (STEM), as well as Cryo-TEM, Energy Dispersive X-Ray Spectroscopy, Energy-Loss Electron Spectroscopy and Image-filtering. These imaging methods are used for ET under the common condition that non-linear effects in the image intensity are avoided^1^,^2^,^3^,^4^. ET attains nanometric resolution in the 3D reconstruction of small volumes and plays a key role in materials science since a volumetric reconstruction can be determinant to understand the structure-property relationship, from the case of complex heterostructures to microelectronic devices^5^. Similarly, the biological function of cellular membranes or fibrous arrays can be evidenced and interpreted by the study of their 3D organization^6^,^7^,^8^,^9^,^10^.

A reliable 3D reconstruction of structures calls for an unbiased, robust, and user independent processing workflow, capable to achieve a tomogram with minimum visual artifacts and even suitable for a quantitative description of the specimen under investigation^11^,^12^. However, such an achievement is prevented by the existing limitations in the experimental process of ET in the TEM, namely the small number of projections that can be acquired without causing a significant beam damage to the specimen, and the inaccessibility of projections at high tilt angles because of the conventional slab geometry of the sample and the limited space for rotation inside the TEM. The restricted angular range is referred as the *missing wedge* and is the main source of elongation artifacts and resolution worsening in the tomograms. From an acquisition perspective, several improvements have been proposed: double tilt-axis or conical tomography, as well as the use of special sample holders^13^,^14^,^15^,^16^,^17^, fast detectors for image acquisition^18^, and sample preparation^19^. Regarding projections processing, remarkable achievements in terms of tomogram refinement have been obtained through iterative reconstruction techniques, namely Simultaneous Iterative Reconstruction technique (SIRT), Algebraic Reconstruction Technique (ART), Ordered-Subset Algebraic Reconstruction Technique (OS-ART) and Discrete-ART (DART)^20^,^21^,^22^,^23^. DART tomography is among the reconstruction methods which exploit some useful prior information about the specimen and is especially suited in materials science, since it relies on the preliminary assumption that only few components with distinct mass densities are combined in the sample^22^. Another example is the use of the symmetry of the shape of a macromolecule, inferred by other techniques, to assist the tomographic reconstruction^24^.

In the same perspective, also the existence of a *sparse* or *compressible* representation of the signal to be retrieved constitutes a significant prior knowledge to be exploited to the extent that the reconstruction problem can be formulated in the framework of Compressed Sensing (CS)^25^,^26^. CS-based reconstruction for ET has been considered in ref. 27 and explored and tested in refs 28 and 29. CS was demonstrated effective in materials science for core-shell heterostructures, concave particles, and nanoporous materials; these examples feature sparsity or compressibility in various domains^30^,^31^. Indeed, sparsity in the native, image gradient, and wavelet domains has been explored for samples with different degree of spatial complexity, as it was the case in ref. 29 for densely packed and even overlapping structures.

Recently, the CS-based approach has been placed under scrutiny with the purpose to improve the tomographic reconstruction of biological samples. The problem of the flimsy structure of the biological samples has to be addressed through an effective acquisition strategy capable to reduce the damage caused by interaction with the electron beam and the reconstruction workflow is required to provide high fidelity reconstruction. Beside the potential performance boost of the CS-approach in pursuing a new application, the method should be thoroughly verified, as pointed out in ref. 32.

The potential of the CS-based approach to the investigation of inherently complex biological structures has been recently approached by ref. 33 and by ref. 34. In these works, the projections of a biological sample imaged in the TEM operated in the Scanning-Transmission imaging mode were used for a CS-refined reconstruction of samples which have been either specifically shaped for the tomographic reconstruction or do not provide significant a-priori information about its structure.

In the present work, a complementary contribution is presented by applying the CS-approach to a slab-shaped biological specimen of collagen fibrils, featuring the important characteristic of a striation with nanometric periodicity. In addition, electron tomography is experimented in the Scanning Electron Microscope (SEM), operated in the low-energy STEM mode and equipped with a specifically designed detector. The potentiality of this approach and its complementary with respect to other solutions are valuable in the context of 3D cellular imaging techniques^35^.

In what follows, the implemented acquisition and processing pipeline are presented: the tailored strategy for the acquisition of the tilt series, the alignment of the acquired projections, and a sparsity-exploiting CS reconstruction with an experimental assessment of the accuracy and robustness of the reconstruction.

## Results

### Tomography in the SEM

The Scanning Electron Microscope is conventionally operated for the visualization of the surface of bulk specimens. In order to explore biological or inorganic structures at various length-scale and resolution, different three-dimensional techniques and experimental approaches, like the so-called *slice-and-view* method, have been developed^36^; where the size of explored volume spans from 100 μm^3^ to 10 μm^3 ^37.

The implementation of the Scanning-Transmission imaging mode in the SEM allows the exploration of the inner structure of samples by means of the energetic electron beam. For a specimen sufficiently thin to provide electron transparency, the intensity of the incoherently scattered electrons was demonstrated to be a monotonic function of sample thickness and mass-density^38^,^39^,^40^.

**Figure 1:**
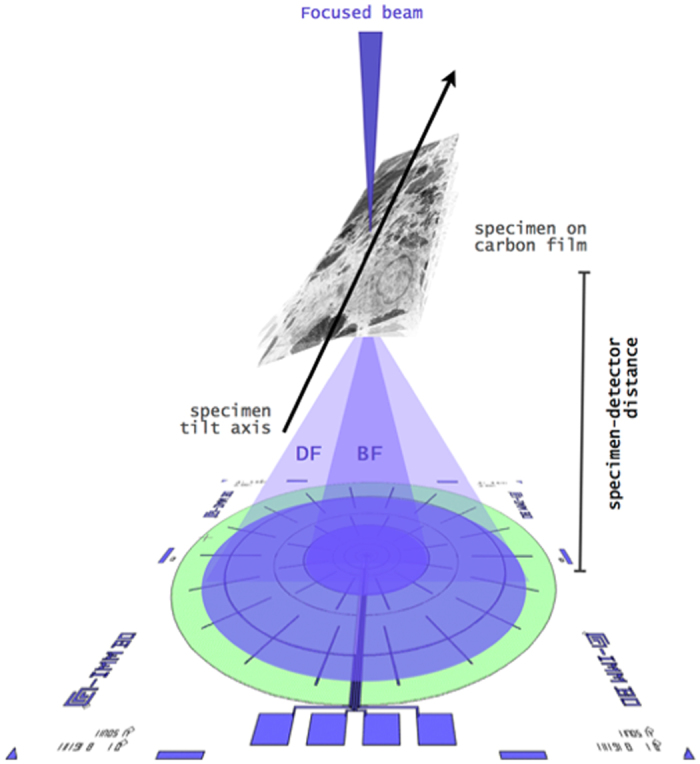
Schematic layout (not to scale) of the STEM projection strategy in the SEM. The specimen rotation axis is normal to the direction of the focused electron beam, and the intensity of both the BF and DF components of the scattered transmitted beam is integrated by the multi-sector detector.

Figure 1 summarizes the projection strategy implemented in the SEM operating in the STEM imaging mode. The thin sample is prepared on a standard TEM grid and supported by an open rotation holder with eucentric capability, in-house designed and fabricated, attached to the SEM stage. The detector is mounted on a separate support, allowing alignment with the center of the beam raster. Beam energy and adjustable specimen-to-detector distance are the basic operative parameters to adapt such experimental set-up to the observation of either thinned organic structures or inorganic nanostructured samples as reported in refs 41 and 42. The focused electron beam of the microscope scans systematically across the specimen while the transmitted electrons are collected by the STEM detector placed below the sample. The tilt-series is acquired by stepwise tilting the sample and recording the corresponding projections. For 20–30 keV incident electrons, the transmitted electron beam suffers from a significant energy loss and scattering in a cone around the forward direction. The transmitted beam could be separated into the Bright-Field (BF) - in the final image the details with lower mass-density appear brighter - and the Dark-Field (DF) - in the final image the details with higher mass-density appear brighter - components and collected by a detector with circular symmetry and independent annular active sectors, allowing one to adapt the detection strategy for the maximum visibility of the details. The STEM image represents a projection of the *mass-thickness* of the structure. Therefore, a series of STEM images at different tilt angles, corresponding to different projection directions, could be used for a tomographic reconstruction.

STEM imaging in the SEM appears promising for ET in the SEM as it takes advantage from some peculiar characteristics of the experimental set-up: the absence of post-specimen lenses allows a nearly-complete collection of the transmitted electrons; the relatively large distance between the sample and the microscope column facilitates the operation of the rotation holder.

The principal constraint for the success of tomography using STEM imaging is to maintain the monotonic variation of the mass-thickness contrast over the whole tilt range. As the projected thickness of the sample, i.e. the measure of the path for the electron beam along the projection direction, increases upon tilting, the scattering angle for the transmitted electrons may be as high as 80° with respect to the forward direction. The imaging system is thus required to collect the entire transmitted signal^39^,^40^,^43^.

**Figure 2:**
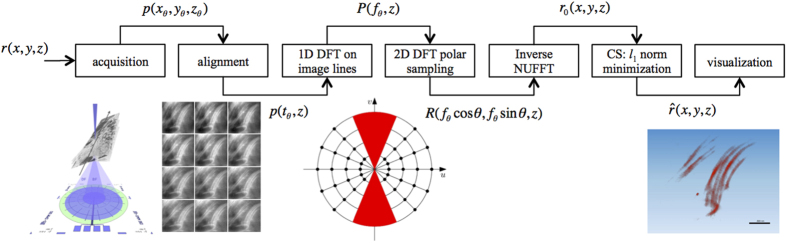
The tomographic reconstruction pipeline from the experimental STEM projections.

The processing pipeline from the acquisitions of the tilt series on the SEM to the tomographic reconstruction and visualization is shown in Fig. 2, while the different steps are described in the following subsections.

### Image Acquisition

**Figure 3:**
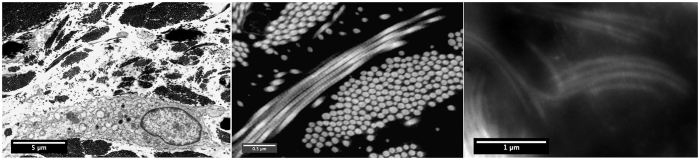
STEM images of a biological sample: (Left) STEM BF panoramic view of cellular structures and collagen fibrils in human derma (Beam energy: 15 kV - collection angle 0–7°). (Center) STEM DF detail of the fibrils, showing a periodical striation along them (Beam energy: 15 kV - collection angle 7–21°) (Right) STEM DF image from the tilt series of the collagen bundle selected for tomographic reconstruction (Beam energy: 30 kV - collection angle 21–45°).

Figure 3 highlights the complex structure of the dermal tissue as revealed by the STEM imaging mode in the SEM. In the BF mode (Fig. 3-*Left*), the lighter parts of the sample are visualized as bright areas, and the darker details correspond to the osmium-stained structures. In this panoramic picture, cellular membranes and circular structures are mixed with bundles of collagen fibrils. A closer view of the specimen shows that the intricate spatial disposition of the fibrils is perceivable, as shown in in Fig. 3-*Center*, where the STEM was operated in the DF mode by activating the outer annular sector of the STEM detector. In DF, the unscattered transmitted electron beam is excluded and the light/heavy details contrast is reversed. Figure 3-*Center* shows that the signal for the fibers is significantly higher than that of the surrounding areas, and the dynamic range allows distinguishing a periodical contrast modulation along the single fibers. This feature fits with the known transverse striation for the collagen fibrils with a periodicity ranging between 64 and 70 nanometers^44^.

The small bundle of collagen presented in Fig. 3-*Right* was selected as the Region of Interest (RoI) for the tomographic reconstruction. Preliminary to the tilt-series acquisition, adaptation of the set-up and verification of the projection requirements were carried out. First, STEM-DF images of the RoI taken at 0° and 50° tilt were compared in order to observe if contrast inversion for the image details occurred upon tilting. By varying the beam energy and the specimen-to-detector distance, the appropriate sector of the detector could be identified. In addition, the histograms of the image intensity allowed setting the 16-bit dynamical range for the tilt-series registration. Eventually, the experimental conditions for the tilt-series acquisition were obtained by increasing the beam energy to 30 keV and selecting the 21°–45° angular range for the collection of the transmitted electrons. This condition allows preserving the monotonic variation of the contrast upon tilting the sample.

### Tilt-compensated alignment

Being it is very unlikely to exactly have the RoI on the tilt axis there is a macroscopic RoI displacement, with respect its dimension, at each rotation step. Therefore, the RoI is taken back to the field of view by manual or semiautomated adjustments on the SEM. The generic acquired projection *p* at an angle *θ* is therefore affected by a displacement error, so that we can write *p*(*x*_*θ*_*, y*_*θ*_*, z*_*θ*_) where the transform from the local reference system (*x*_*θ*_*, y*_*θ*_*, z*_*θ*_) to the common reconstruction reference system must be estimated for each tilt angle and, preliminary to the reconstruction, the projection misalignments must be corrected^13^. Given the orthographic projective acquisition geometry, an affine camera model could be adopted^45^ for the displacement estimation. However, the adoption and parameter estimation of this general model can be avoided and an equivalent solution can be found by the exploitation of the *a-priori* information we have on the acquisition geometry of the experimental set-up. In this case, we assumed that the tilt axis is aligned to the *z*-axis and orthogonal to the projection direction; the angular rotation step is also known and the magnification factor is constant. The verification of the assumption on the direction of the tilt axis will be discussed in the following sections, based on the evidence of artifacts in the reconstructed tomograms. Cosine stretching in the direction perpendicular to the rotation axis can be applied to the target projection image to estimate, by image cross-correlation, its alignment with respect to a reference image^46^. This alignment technique has been adopted in this work. Alternative accurate methods have been proposed based on the insertion of artificial markers within the sample^40^. Also markerless approaches have been proposed, usually based on correlation tracking of salient image features on small windows^41^,^42^. These techniques can however suffer from the presence of repeated structures in the sample (as it is the case here) or by radiometric contrast changes that may occur especially at the extremities of the SEM tilt series, as described in^43^.

**Figure 4:**
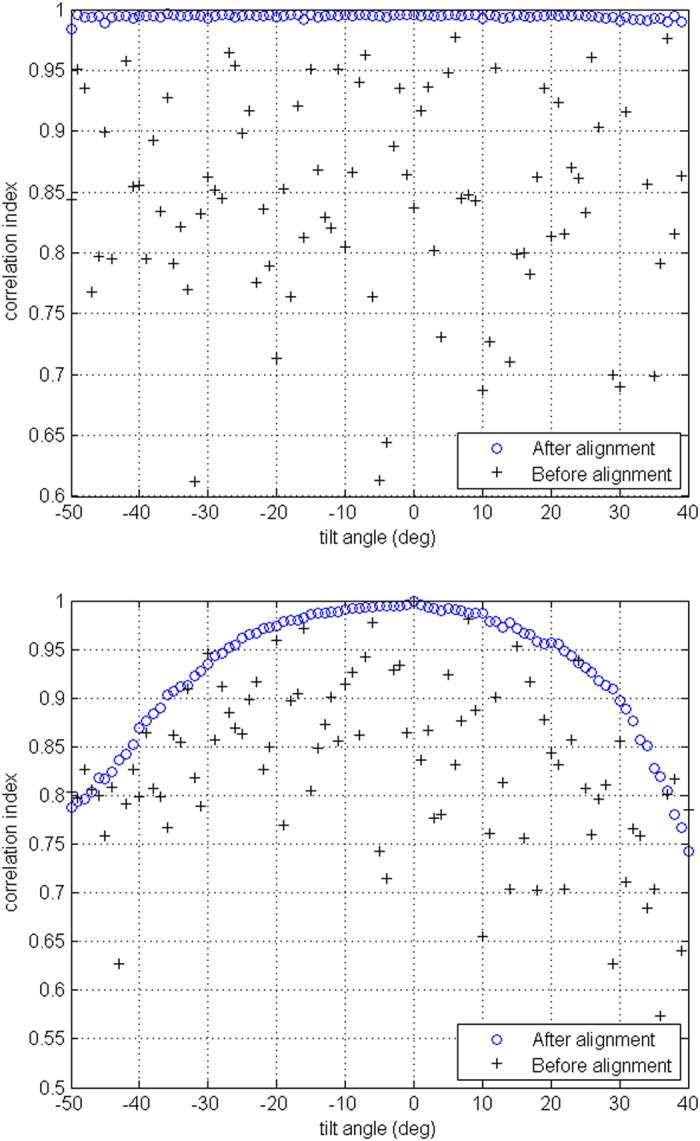
Cross correlation for the projections before and after the alignment. (Top) Values for consecutive images, (Bottom) Values calculated with the projection at 0° as fixed reference.

The alignment performance is presented in Fig. 4, which reports the comparison between the correlation index calculated across consecutive images (top) or considering the projection at 0° tilt as fixed reference (bottom). In the top graph, the values tend towards unity, with few exceptions, which could be ascribed by slight defocus of the electron probe. In the bottom graph the values of correlation index decrease symmetrically and regularly for negative/positive tilt, owing to the significant difference between projections taken at increasingly higher angle (the smoothness of the curve is of significance in this case).

### Reconstruction initialization

Aiming to implement a tomographic reconstruction of the volume of interest *r*(*x, y, z*), thanks to the orthographic nature of the projections and to their alignment, the problem can be decomposed in a slice-by-slice reconstruction on the stack of lines of the projection images grouped with respect to the discrete set of tilt angles *θ* (we adopt a light notation by avoiding discretization indexes).

Projections can be then represented as *p*(*t*_*θ*_*, z*) images with (*x, y, z*) = (*t*_*θ*_ cos *θ*, *t*_*θ*_ sin *θ, z)*. Hereinafter, we also just drop the *z* coordinate and follow what happen for a generic reconstruction slice. The samples of the 1D (discrete) Fourier transforms *P*(*f*_*θ*_) of the set of aligned projections *P*(*t*_*θ*_) can be seen as samples of the 2D Fourier transform of the volume slice *R*(*u, v*) = *f*{*r*(*x, y*)}. In fact, according to the central (Fourier) slice theorem^47^, these samples are arranged such that *R*(*u, v*) = *R*(*f*_*θ*_ cos *θ*, *f*_*θ*_ sin *θ*) This entail the typical radial sampling configuration (with a *missing wedge* in our case) that is the starting point for possible inversions. An effective inversion (backprojection) of the radial sampling pattern can be done using the Non-Uniform FFT (NUFFT) implementation described and available in ref. 48. Despite the presence of reconstruction artifacts (caused by the possibly limited number of projections and by the missing wedge) the NUFFT inversion has been proven to be able to offer a useful initialization and to effectively operate in a CS based reconstruction refinement^20^.

### Sparsity based reconstruction

The Compressed Sensing framework^25^,^26^ offers powerful tools for signal reconstruction starting from undersampled measurements under the conditions that the data are sparse in some representation domain and that the sensing (sampling) operator (matrix) and the domain representation operator (basis) are incoherent each other. Here we are interested to verify, more from a practical than from a theoretical point of view, that a CS formulation is able to provide accurate reconstruction in our ET setting. CS has been already demonstrated to be compatible with Magnetic Resonance^49^ and X-ray Computed Tomography^20^,^48^ reconstructions, and to be a powerful tool in ET problems^28^,^29^,^30^,^48^. As in previous studies, we are interested to verify accuracy and robustness of our CS-based reconstruction pipeline with the novelty here of considering a biological sample and data acquired with the SEM. The experimental sample has been selected such that some structural parameters (both visual and quantitative) are known in advance. It is thus possible to measure and verify them on the reconstructed data and to assess the robustness of the reconstruction against different acquisition and projections shortage profiles. We are interested in assessing the sparsity-based refinement with respect to different domain operators. In particular here we consider the gradient domain (total variation operator) and the image domain (identity matrix). The latter is because the DF acquisition mode in the SEM, by highlighting the collagen fibers with respect to the background, can be seen as fostering sparsity in the native image domain.

Therefore, similarly to^29^, we consider the following refinement formula


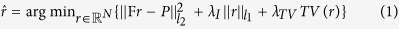


where P is the vector of the transformed projections and F is the so called undersampled Fourier operator^29^,^49^ which, in our case, corresponds to a NUFFT operated on the frequency radial sampling pattern masked by the missing wedge (see Fig. 2). Discussions on the applicability of such a kind of sampling (sensing) pattern in the context of CS, where random sampling is proposed in most cases to be desirable from a theoretical point of view, have been done in several works. In particular, this was first shown in ref. 50, then further explored in ref. 51, discussed in ref. 49 and applied in ref. 29. From these works, non-uniform radial sampling in the Fourier domain (or k-space for MRI) demonstrated its feasibility and effectiveness, de-facto contributing to determine CS to be a suitable technology for tomographic reconstructions and specifically for ET. In (1) the first term to minimize is the tolerance computed in the Fourier domain, the second and third terms impose the sparsity in direct image domain (*I*) and in the gradient domain (by means of the total variation norm *TV* of the spatial gradient of *r*) through the Lagrange multipliers *λ*_*I*_ and *λ*_*TV*_ respectively.

Working with direct image domain sparsity is twofold interesting in our case because: (a) the sample preparation described in the section Methods tends to enhance structural elements (e.g. membranes, fibrils, …) in the images so that the acquisition of tilt series and the related ET reconstructions can be suitably focused on these structures of interest (see Fig. 3) and benefit of a natural presence of a low energy (dark) and/or uniform background; (b) as experimentally reported in refs 29,30 sparsity in the image domain is helpful in reducing the artifacts due to the missing wedge, which are prominent in backprojection-based and iterative reconstructions.

The optimal solution is obtained by a conjugate-gradient-descend minimization algorithm described and available in ref. 49.

### Tomogram visualization

**Figure 5:**
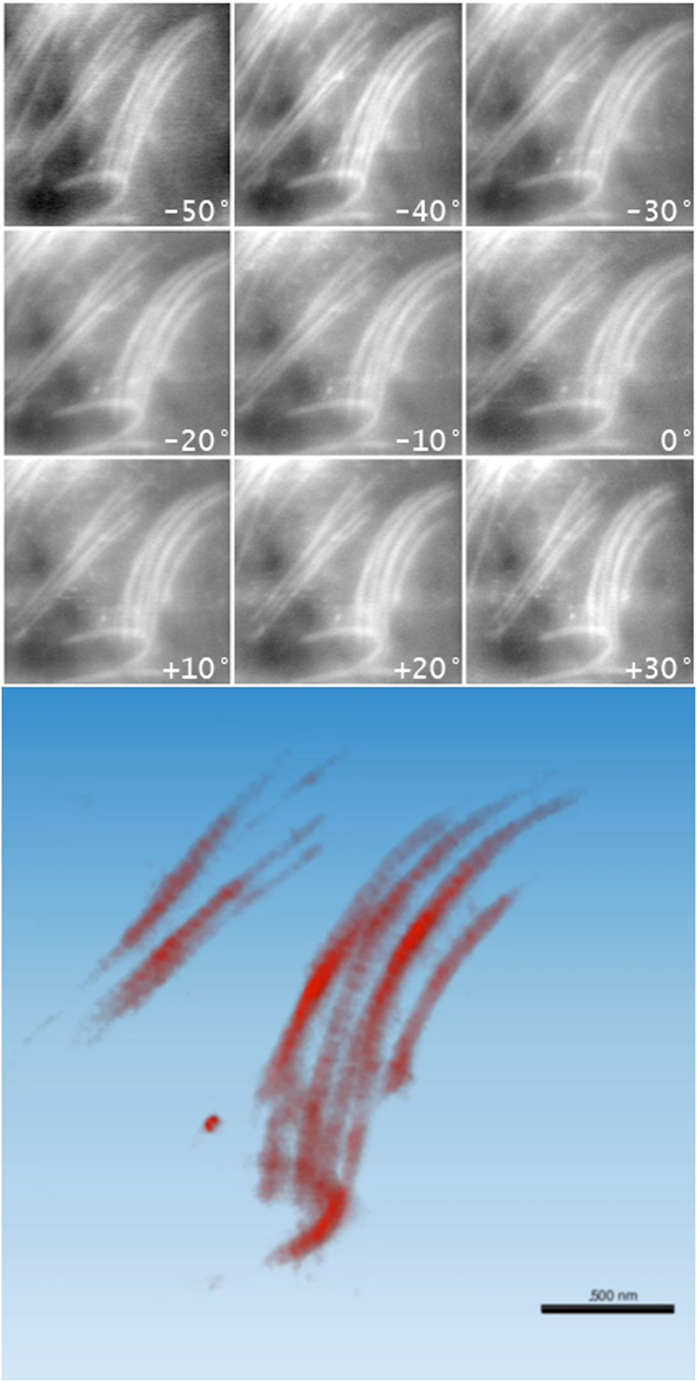
(Top) Some of the STEM DF images from the tilt-series. The tilt angle is reported for each projection, being the electron beam perpendicular to the specimen at 0°. The z-axis corresponds to the direction of the electron beam for the reconstructed tomogram. (Bottom). A view rotated by an angle of 20° around the horizontal x- axis of the tomogram of the collagen bundle refined by CS. The typical striation for the collagen fibrils is visible indicating that an appropriate resolution was achieved.

Nine images from the aligned tilt-series of 91 projections are presented in Fig. 5-*Top*. The region of interest is composed by two collagen bundles, which have been reconstructed and visualized in Fig. 5-*Bottom*. *Voxel*-*size* corresponds to 7 nm and the reconstructed volume measures about 8 μm^3^. The rendering parameters showcases the single fibrils for the two bundles and the surrounding embedding matrix was excluded from the visualization, owing to the absence of significant details.

**Figure 6:**
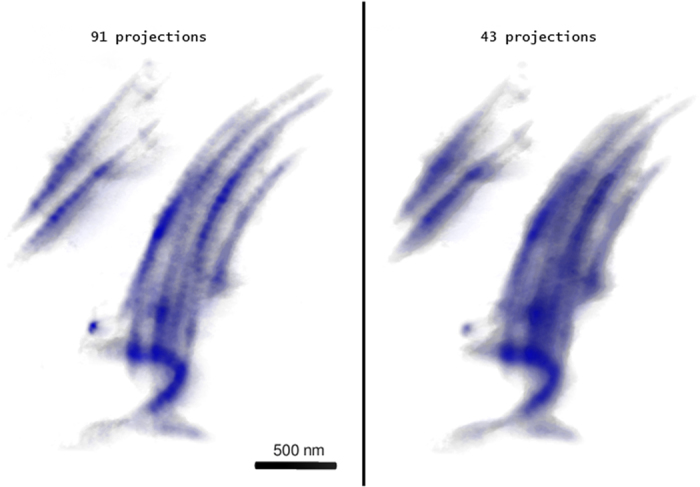
Comparison between OS-ART reconstructions of the fibril bundle using 91 (Left) or 43 (Right) projections. The visibility of the striation is significantly reduced in the second tomogram.

Figure 6 shows the reconstruction obtained with the iterative ordered-subset simultaneous algebraic technique (OS-ART) implemented in TomoJ^23^,^52^. The reconstruction with all of the 91 available projections from the experimental tilt series is complete and detailed, however the reduction to 43 of the number of projections determines a significant decrease in the visibility of the striation.

The number of projections available for the reconstruction has a great effect on the accuracy and resolution of the tomogram, and the capability to retrieve the significant details from a limited number of projections is especially important in the biological field.

It can observed that the CS-refined tomogram presented in Fig. 5 compares positively with the OS-ART reconstruction; therefore, the potential of the CS approach to preserve the details upon decrease of the number of projections becomes of primary interest here and will be addressed in the following sections.

### Sparsity weighting parameters adjustment

**Figure 7:**
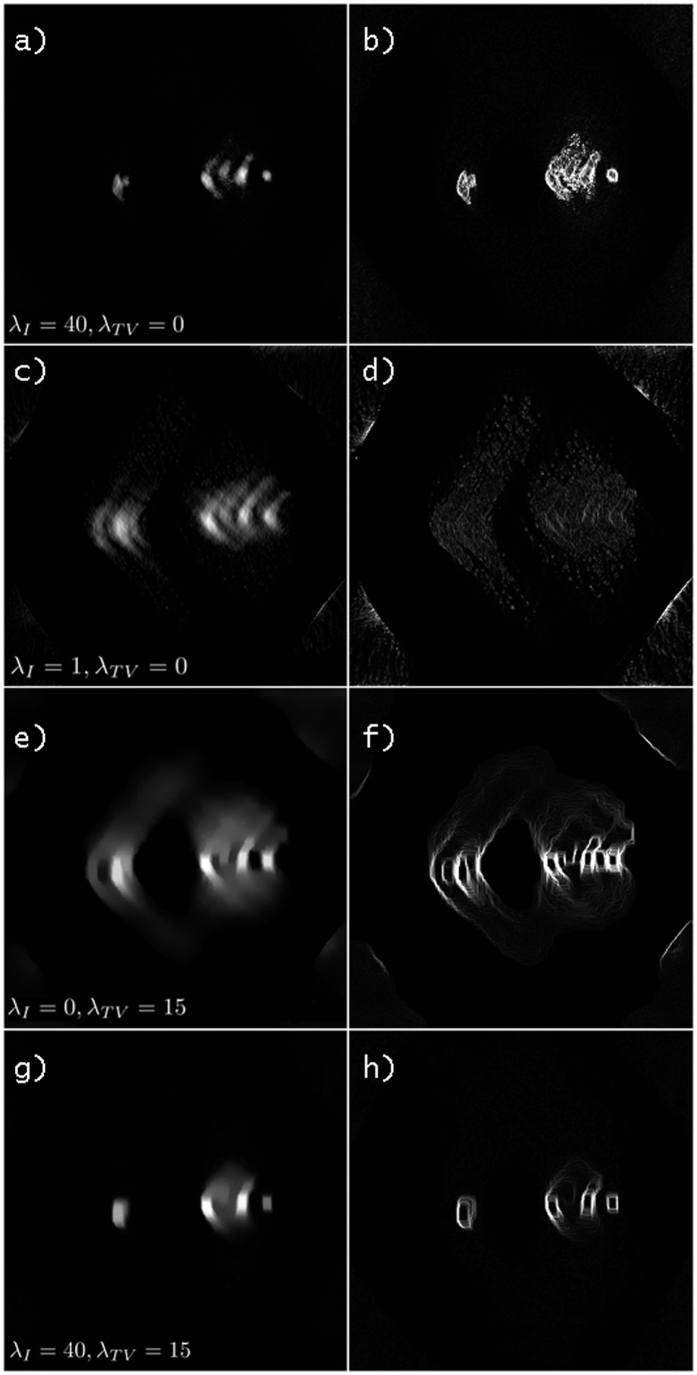
Dependence of the reconstruction on the multipliers *λ*_*I*_ and *λ*_*TV*_. (**a,c,e,g**) A representative slices of the tomogram, (**b,d,f,h**) the corresponding absolute gradient image of the selected slices. The values of *λ*_*I*_ and *λ*_*TV*_ for the slices are also reported. The intensity scale of the images have been adapted for the best visualization of the blurring or the edge enhancement of the structures. The streaks are attributed to the missing wedge in the sampling scheme, and the images also highlight a curved artifact, which is attributed to a slight deviation of the tilt axis from the assumed direction.

According to the chosen expression to be minimized, the values for the *λ*_*I*_ and *λ*_*TV*_ parameters have to be properly determined. Deviations from the optimal values results either in blurring of the tomogram or excessive enhancement of edges and noise-like artifacts^29^. According to the literature on the application of CS-ET to inorganic samples, this detrimental effect is ascribed to the underestimation or overweighting of the sparsity in the gradient image domain; moreover, promoting sparsity in the direct image domain may result beneficial. Figure 7 shows the comparison between representative slices of the tomograms computed for different *λ*_*I*_ and *λ*_*TV*_ values; the corresponding gradient images are also presented, confirming that the expected artifacts arise from the unbalanced application of the *λ*_*I*_ and *λ*_*TV*_ parameters. In the biological system under investigation, optimization occurs for a vanishing *λ*_*TV*_ and a preponderant influence attributed to the sparsity term *λ*_*I*_ in the image domain: this differs from the cases reported in application of CS to materials science, and could be ascribed to the process of specimen preparation and visualization in the STEM system. The preparation, to be regarded as conventional in the TEM microscopy of biological samples, enhances the visibility of the embedded structure’s details: the localization of heavy osmium atoms in correspondence of fibrils and membranes intensifies the contrast with respect to the surrounding matrix composed by light elements (C, O, and H). In the CS framework, this preparation could be considered as an increase of image coefficients for the significant biological details with respect to the embedding matrix, concurrently promoting the sparseness of the image.

The slices of the tomogram proved also useful in observing the impact of the missing wedge on the reconstruction and to recognize the presence of curved artifacts in the reconstructed mass density, which could be ascribed to an uncorrected misalignment of the tilt-axis with respect to the assumed one.

### Visual and quantitative evaluation of the reconstructed collagen bundle

The spatial disposition of the two bundles is properly reconstructed and the striation of the collagen fibrils is clearly visible, demonstrating that the achieved resolution is adequate to reveal the finest details present in the specimen. The opportunity to assess the accuracy of the reconstruction by exploiting an existing periodical feature, of a size comparable to the needed resolution for the tomogram, is uncommon in the biological field where biological objects exhibit variability and complexity in the constituents’ structure.

The optimization of the parameters and the importance of the number of projections used for reconstruction is also observed by calculating the *projection error ε*_*p*_ over the reconstructed tomogram; this comprehensive figure is defined as:


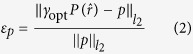


where, *p* is a discrete projection operator, *p* is the set of the experimental projections (in vectorial or sinogram form) and, as suggested in^8^, an optimized scaling factor *γ*_opt_ = min_*γ*_||*γp* 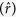 − *p*||*l*_*2*_ is to be adopted to compensate for an intensity range modification possibly occurring on the reconstruction^49^.

Accordingly to the evident reduction in image artifacts, the projection error for the reconstructed tomograms decreases upon optimization of the weighting parameters and reaches a minimum value of 0.1034 for the best tomogram.

**Figure 8:**
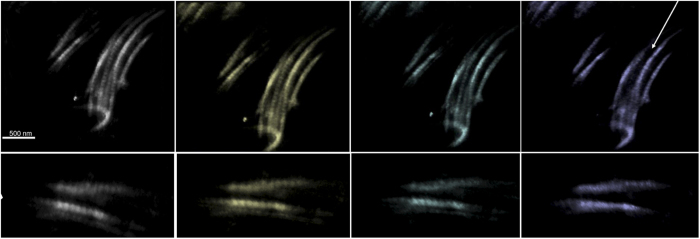
(Top) Comparison between tomograms obtained from a different number of projections. Visualization of (x,y) planes at 0° tilt. From left to right: 91, 46, 31, 16 projections. The white arrow in the last tomogram indicates that a collagen fibril is missing from the visualization (Bottom) Four detailed views from the above tomograms show the decreasing resolution in presenting the striation of collagen.

Table 1, together with the visual comparison presented in Fig. 8, shows that the value of *ε*_*p*_ increases as the number of projections used for the reconstruction is decreased. It turns out that a significant reduction from 91 to 46 and even down to 31 of the number of projections could be applied to compute the tomogram without significant information loss. Fewer projections would result in higher error and in the blurring of image details.

As marked by the white arrow in the upper-right panel of Fig. 8, one fiber of the collagen bundle and the characteristic striation of collagen are not longer visible in the tomogram obtained from 16 projections.

According to the definition given by Crowther, the tomogram resolution is governed by the number of available projections, which are assumed to be noise-free, perfectly-aligned, and covering the entire angular range^13^. Expected values of for the resolution are reported in Table 1. However, the spectral signal-to-noise ratio, a residual projection misalignment, and the missing wedge introduce an additional resolution worsening. In the reconstruction of collagen, the known periodicity of the striation is helpful in the evaluation of the resolution. For the sake of a numerical estimation, the resolution turns out to be better than 60 nm, a value comparable to the ideal Crowther estimate of 24 nm for 91 projections. Despite the rapid increase of the nominal resolution, which equals the periodicity of collagen when 31 projections are considered, the corresponding CS-assisted reconstruction still exhibits a clear contrast modulation along the fibrils (see Fig. 8).

An independent random shift for the images has been introduced to determine the impact of the alignment method on the projection error. Table 1 reports the projection error calculated from different tomograms. The maximum displacement of 2–3 pixels corresponds to 15–21 nm and is the cause of a significant worsening of the tomogram quality. Differently, the last values for the CS reconstruction have been obtained by restricting the cross-correlation alignment procedure to only the projection used for reconstruction. In the case, the increase of the error was restrained. This is an indication that obtained alignment is adequate for the reconstruction task, and that the number of projections used for reconstruction plays the fundamental role.

For comparison, the projection error calculated from Fourier-Back-Projection (FBP) reconstructions are also reported.

It turns out that the alignment method and the reconstruction workflow are so functional that a significant reduction of the number of projections needed for reconstruction could be operated. This indication is important for the improvement of the acquisition strategy as the reduction of projection opens the possibility to observe beam-sensitive specimens and to perform the acquisition in shorter times.

The capability of the CS approach to assist the reconstruction could be used to harmonize the acquisition strategy with the reconstruction workflow. A more effective scheme could be envisaged by taking into account the CS approach. The adopted sampling scheme, based on a conventional single-axis rotation, could be improved to meet the sampling randomness and incoherence required by CS. The peculiar implementation of tomography in the SEM opens up the possibility to implement projection schemes different from the fixed, single-axis rotation used in this study and derived from the operation in the TEM.

## Discussion

A tailored strategy for the scanning-transmission imaging mode has been implemented in the scanning microscope, resulting in a low-energy innovative counterpart of the traditional approach to electron tomography. This approach may improve the present procedure for site-selective cryo-tomography of biological specimens as the final step of image acquisition could be performed in the FIB/SEM microscope, avoiding the transfer of the specimen in the TEM^53^.

The experimental setup was proved effective in acquiring projections of a representative biological sample, and the reconstruction assisted by CS was able to overcome the limitations arising from few projections and missing wedge in the sampling scheme, while showcasing the finest details in the reconstruction of collagen fibrils.

In the case of a significant reduction of experimental projections, a restrictive condition for iterative reconstruction methods, CS preserved the accuracy in reconstruction.

This investigation indicates a favorable improvement for the acquisition strategy in the capability of CS to promote the tomographic reconstruction of a sample, which poses the typical challenges for the biological field. The technique is expected to provide the reconstruction of volumes up to 20 μm × 20 μm × 0.7 μm, composed by light elements and with thickness controlled by the fine microtome slicing.

The suitability of CS for accurate and robust refinement of the reconstruction applied in this novel configuration shows a potential of wide application in life sciences and biological imaging as well as for the visualization of other light materials such as polymers, carbon nanotubes, nanostructured crystals.

## Methods

### Experimental set-up

A ZEISS LEO 1530 SEM was operated at 30 keV beam energy for the highest resolving power, specimen penetration, and detail contrast. The stage was modified to rotate the specimen and govern the collection angular range. The size of the focused electron beam (about 1 nm) ultimately limits the resolution for the images.

The STEM imaging mode is already implemented in modern SEMs, and all the principal manufacturers provide a detector suitable for the purpose. The use of STEM for tomography requires however an acquisition system capable of collecting the transmitted electron over a large and adaptable angular range. The STEM detector and the corresponding signal conditioning system were fabricated with optimized design and performance in order to comply with the significant variations of projected thickness for the specimen occurring in the tomographic application. The layout of the detector reported in Fig. 1 shows its basic features: four independent sectors with high and constant detection efficiency over the 5–30 keV energy range. Beam energy and specimen-detector distance are the additional operative parameters to govern the collection angles.

For the acquisition of the tilt series, the sample was stepwise rotated by 1° degree from −50° to +40° and the corresponding STEM projections were acquired with 30 keV beam, 100 nm dwell time, approx. 200 pA beam current, at 40.000× magnification, corresponding to a 7.35 nm pixel-size; 16-bit 1024 × 768 images were recorded.

### Preparation of the biological sample

The preparation of the collagen specimen from human dermal tissue was carried out following the standard routine used for transmission electron microscopy. Samples were fixed in 2.5% glutaraldehyde and 1% osmium tetroxide, dehydrated, and embedded in Spurr resin. Unstained sections were prepared by ultramicrotomy with 0.7 μm thickness^40^. The relatively large thickness of the section and conventional suspending system on *Formvar* coated copper grids prevented the projections of the sample from being accessible at angles higher than 60°.

The preparation also addresses the sample’s capability to withstand the electron irradiation: as the electron dose in the low-energy STEM mode is relatively small, it was observed that a series of about 100 images could be acquired with no significant specimen shrinking and damage.

### Software for processing and reconstruction

The processing of the tilt series and the calculation of the tomogram was carried out in *Matlab* (specific adopted routines and packages available from scientific works will be referenced in the following sections). *Chimera* software has been used for the tomogram visualization along with *ImageJ* and *TomoJ*^52^,^54^.

## Additional Information

**How to cite this article**: Ferroni, M. *et al*. Biological application of Compressed Sensing Tomography in the Scanning Electron Microscope. *Sci. Rep.* **6**, 33354; doi: 10.1038/srep33354 (2016).

## Figures and Tables

**Table 1:** Summary of the values for the projection error calculated for CS-based reconstructions with different number of projections.

#proj	91	46	31	23	16	11
CS	*alignment using all of the 91 projections*					
	0.103	0.111	0.111	0.119	0.129	0.152
	0.138	projections shifted by ±2 pixels at most				
	0.165	projections shifted by ±3 pixels at most				
	*alignment using only the considered projections*					
		0.125	0.118		0.156	0.165
FBP	*alignment using all of the 91 projections*					
	0.254	0.253		0.256		0.266
*d* (nm)	24	48	71	96	137	200

The projection error for reconstructions based on FBP and the nominal resolution *d* are also reported.
